# Mitochondria and Caspases Tune Nmnat-Mediated Stabilization to Promote Axon Regeneration

**DOI:** 10.1371/journal.pgen.1006503

**Published:** 2016-12-06

**Authors:** Li Chen, Derek M. Nye, Michelle C. Stone, Alexis T. Weiner, Kyle W. Gheres, Xin Xiong, Catherine A. Collins, Melissa M. Rolls

**Affiliations:** 1 Huck Institutes of the Life Sciences, and Biochemistry and Molecular Biology,The Pennsylvania State University, University Park, Pennsylvania, United States of America; 2 Molecular, Cellular and Developmental Biology, University of Michigan, Ann Arbor, Michigan, United States of America; The University of Western Australia, AUSTRALIA

## Abstract

Axon injury can lead to several cell survival responses including increased stability and axon regeneration. Using an accessible Drosophila model system, we investigated the regulation of injury responses and their relationship. Axon injury stabilizes the rest of the cell, including the entire dendrite arbor. After axon injury we found mitochondrial fission in dendrites was upregulated, and that reducing fission increased stabilization or neuroprotection (NP). Thus axon injury seems to both turn on NP, but also dampen it by activating mitochondrial fission. We also identified caspases as negative regulators of axon injury-mediated NP, so mitochondrial fission could control NP through caspase activation. In addition to negative regulators of NP, we found that nicotinamide mononucleotide adenylyltransferase (Nmnat) is absolutely required for this type of NP. Increased microtubule dynamics, which has previously been associated with NP, required Nmnat. Indeed Nmnat overexpression was sufficient to induce NP and increase microtubule dynamics in the absence of axon injury. DLK, JNK and fos were also required for NP. Because NP occurs before axon regeneration, and NP seems to be actively downregulated, we tested whether excessive NP might inhibit regeneration. Indeed both Nmnat overexpression and caspase reduction reduced regeneration. In addition, overexpression of fos or JNK extended the timecourse of NP and dampened regeneration in a Nmnat-dependent manner. These data suggest that NP and regeneration are conflicting responses to axon injury, and that therapeutic strategies that boost NP may reduce regeneration.

## Author Summary

Unlike many other cell types, most neurons last a lifetime. When injured, these cells often activate survival and repair strategies rather than dying. One such response is regeneration of the axon after it is injured. Axon regeneration is a conserved process activated by the same signaling cascade in worms, flies and mammals. Surprisingly we find that this signaling cascade first initiates a different response. This first response stabilizes the cell, and its downregulation by mitochondrial fission and caspases allows for maximum regeneration at later times. We propose that neurons respond to axon injury in a multi-step process with an early lock-down phase in which the cell is stabilized, followed by a more plastic state in which regeneration is maximized.

## Introduction

The ability of neurons to survive injury, misfolded proteins, hypoxic stress and other deleterious conditions allows the nervous system to function for a lifetime without large-scale production of new neurons. Neuronal survival strategies buy the cells time to maintain or regain function. For example, neurons may remain non-functional for weeks, months or years after axonal trauma. Their survival allows axon regeneration to take place, and eventually, if an appropriate target is reached, the cells may again function.

Preconditioning is a transient survival strategy triggered by a stressful, but sublethal, event. For example, when blood flow to a region of the brain is transiently reduced, the effects of a subsequent ischemic stroke are not as severe [1, 2]. Tissue-level preconditioning seems to have an immediate phase, and then a longer-term transcription-dependent phase [2, 3] and is proposed to be a very general stress response mechanism.

Preconditioning has also been described at a single cell level. In Dorsal Root Ganglion (DRG) neurons, severing the peripheral axon enables the central axon for regeneration [4]. The initial peripheral lesion triggers transcriptional changes in the cell body that are proposed to facilitate subsequent regeneration of the central axon [5, 6]. In *Drosophila* models of conditioning lesion in sensory and motor neurons, axon severing turns on a stabilization pathway that is measured by resistance to degeneration after a subsequent injury [7, 8]. This single cell neuroprotection (NP) requires dual leucine zipper kinase (DLK) [7] and c-Jun N-terminal Kinase (JNK) [8]. DLK is a MAP kinase kinase kinase, and JNK is the downstream MAP kinase, which play central roles in the regulatory cascade that initiates axon regeneration in nematodes, flies and mammals [9–12]. DLK/JNK are therefore implicated in regulation of both axon regeneration and preconditioning or NP in response to axon injury.

Using the *Drosophila* sensory neuron model for preconditioning, we investigate the effectors mediating NP downstream of DLK/JNK, and the relationship between NP and axon regeneration. One hallmark of NP is a dramatic increase in microtubule dynamics [8], a response that has also been seen in mammalian neurons [13]. Mitochondria have been suggested to play a central role in brain preconditioning [14], and are important for axonal stability in C. elegans [15] and in many systems the Wallerian degeneration slow (Wlds) protein seems to act through mitochondria to stabilize axons [16–19]. We therefore started by investigating the role of mitochondria in NP. Surprisingly, we found that, rather than promoting NP, mitochondria have an inhibitory role in this process, and caspases share this negative regulatory role. Moreover, although regeneration and NP are downstream of the same kinase cascade, NP antagonizes regeneration. These results are unexpected, but fit together into a multi-step model of axon injury responses downstream of DLK/JNK.

## Results

### Reducing Miro and milton increases axotomy-induced neuroprotection

**Fig 1:**
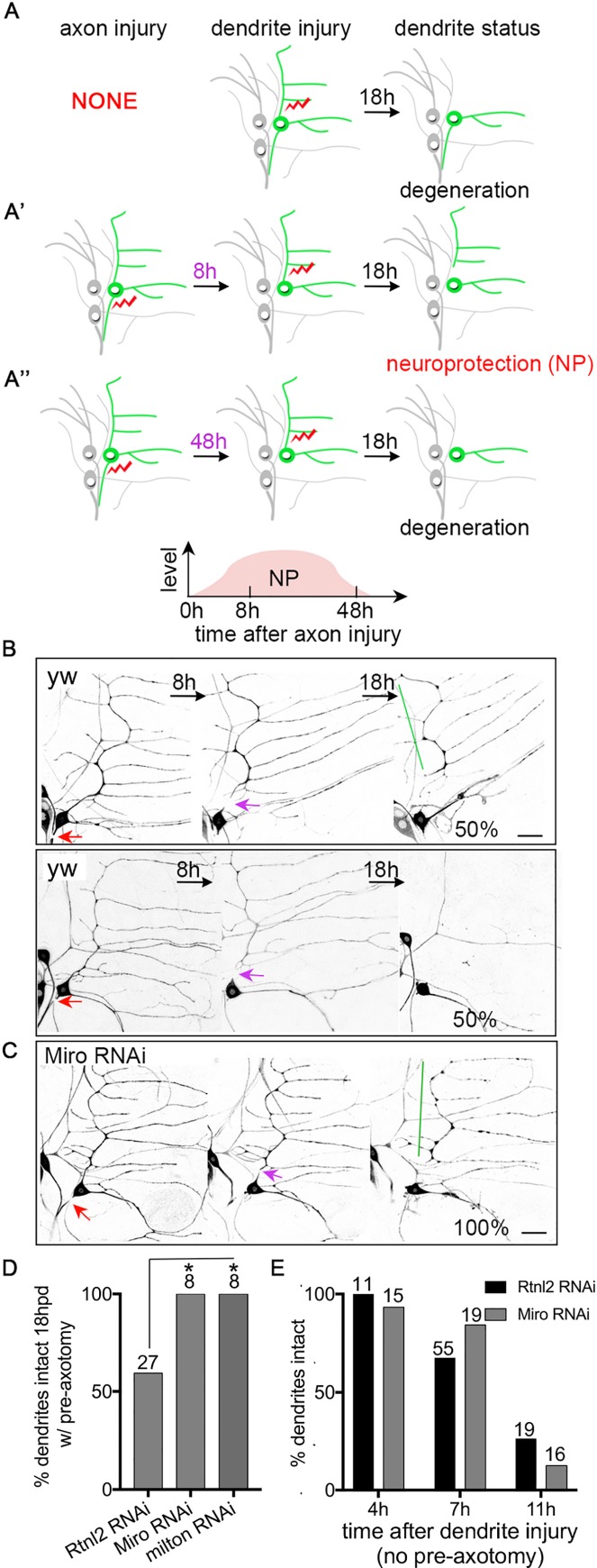
Reducing mitochondria in dendrites increases axotomy-induced neuroprotection. (A-A”) A schematic of the axotomy-induced neuroprotection/NP assay is shown. (A) Without pre-axon injury, dendrites degenerate within 18h after injury. (A’) An axon injury 8h prior to dendrite injury induces NP so that dendrite degeneration is delayed; this timeline is the standard one used to assay NP throughout. (A”) When 48h elapses between the axon injury and dendrite severing, very little NP is observed and most dendrites are gone 18h after they are removed. Laser-induced injury is indicated by red lightning bolts. The ddaE neuron is drawn in green and other neurons labeled by 221-Gal4, which was used in most experiments to drive expression, are drawn in grey. (B and C) The NP assay as illustrated in Fig 1A’ was performed in wide-type (yw indicates control neurons that do not express an RNAi hairpin, and Rtnl2 indicates neurons that express a control RNAi hairpin) and Miro RNAi neurons. Neurons were labeled with EB1-GFP under the control of 221-Gal4. In control conditions half the neurons have a dendrite that remains at 18h, and (top row) and half have a fully degenerated dendrite (bottom row in B). Red arrows are the site of axon injury; purple arrows mark the site of dendrite injury. Green lines indicate stabilized dendrites. The scale bar is 20 μm. (D) Quantification of NP is shown. The number of neurons analyzed for each genotype is indicated above the bars. A Fisher’s exact test was used to determine statistical significance. Rtnl2 RNAi was used as a control as it targets a non-essential gene for which we have never observed phenotypes. * p<0.05. (E) Dendrites in ddaE neurons expressing EB1-GFP and control or Miro RNA hairpins were severed without prior axon injury. The presence of intact dendrites (no breaks in continuity) was scored at 4h, 7h and 11h after dendrites were severed. The numbers above the bars are the numbers of cells analyzed; one cell per animal.

In *Drosophila* sensory neurons, severing an axon with a pulsed UV laser stabilizes the cell such that if a dendrite is later removed its degeneration is delayed [8]. Dendrites normally degenerate completely within 18h (Fig 1A). However, when axons are damaged 8h prior to dendrite injury, the severed dendrites are stabilized and take more than 18h to fragment [8]. Stabilization is maximal at 8-24h and tapers off at 48h after axon injury [8] (Fig 1A). The timing of this stabilization correlates with a dramatic increase in the number of growing microtubules, and this increase in microtubule dynamics is required for stabilization [8].

To assess the role of mitochondria in axotomy-induced stabilization or NP, we depleted mitochondria from dendrites using RNAi-mediated knockdown of the mitochondrial Rho-GTPase Miro, which is required for mitochondrial transport in neurons [20, 21]. We have previously shown that Miro RNAi reduces the number of mitochondria in dendrites of ddaE neurons [22]. Because the NP assay uses the speed of dendrite degeneration to probe stability, we tested whether reduction of mitochondria would affect dendrite degeneration itself, without prior axon injury. We previously demonstrated that small regions of dendrites with no mitochondria degenerate with normal timing [22]. Here, we severed the whole dendrite with normal numbers of mitochondria (wild-type, WT) or reduced mitochondria (Miro RNAi) and assayed degeneration at different times after severing. The time course of degeneration in neurons expressing a control (Rtnl2) RNAi or Miro RNAi was similar (Fig 1E) with a few cells starting to degenerate at 7h after dendrite injury, and most cells degenerating by 11h after severing. In the standard NP assay we sever an axon, wait 8h, then sever a dendrite. Dendrite degeneration in this assay is scored 18h after dendrite severing, so the time course we used to assay dendrite degeneration alone (4h, 7h, 11h) was much finer and should have picked up any small differences in speed of degeneration without prior axon injury.

As Miro reduction did not change the timing of dendrite degeneration, we performed the NP assay in control and Miro RNAi neurons. This assay was performed as diagrammed in Fig 1A’. In control neurons, axon injury 8h before dendrite severing results in about 50% of dendrites remaining at 18h (Fig 1B and 1D and [8]). In Miro RNAi neurons, NP was increased and 100% of dendrites remained at the 18h timepoint (Fig 1C and 1D). RNAi targeting milton, which recruits Miro to mitochondria [20, 21], also increased NP (Fig 1D). These results suggest that normal mitochondrial trafficking or dynamics limits injury-induced NP.

### Drp1-mediated mitochondrial fission inhibits neuroprotection after axotomy

**Fig 2:**
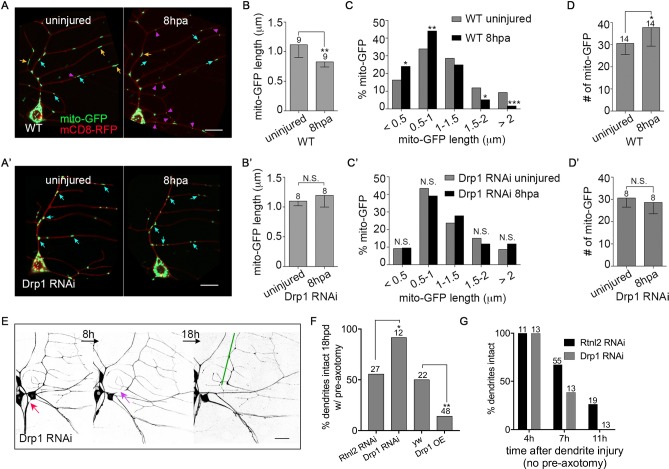
Axotomy-induced mitochondrial fission inhibits neuroprotection. (A and A’) Representative images of dendritic mitochondria in control (A) and Drp1 RNAi (A’) neurons before and 8h post axon injury (hpa) are shown. mito-GFP and mCD8-RFP were coexpressed in ddaE neurons under the control of 221-Gal4 in order to visualize mitochondria and the cell membrane, respectively. Orange, blue and magenta arrows indicate long (>1.5 μm), medium (1–1.5 μm) and short (<1 μm) mitochondria respectively. Scale bars are 10 μm. (B-D’) The length, length distribution, and total number of mitochondria in control (B-D) and Drp1 RNAi neurons (B’-D’) before and after axon injury were measured. Statistical significance was determined using a Fisher’s exact test (C and C’), or a t test (B, B’, D and D’). * p<0.05, ** p<0.01, *** p<0.001, N.S. not significant. Error bars represent SD. (B, B’, D and D’) The numbers of neurons analyzed are indicated above the bars. (C) 227 and 229 mitochondria from 9 neurons were analyzed for uninjured and 8hpa, respectively. (C’) 173 and 169 mitochondria from 8 neurons were analyzed for uninjured and 8hpa, respectively. (E) The NP assay was performed in Drp1 RNAi neurons labeled with EB1-GFP. Red arrows indicate the site of axon injury and purple arrows, dendrite injury. Green lines mark stabilized dendrites. Scale bar, 20 μm. (F) Quantification of NP is shown with control data from Fig 1D for comparison. * p<0.05 and **p<0.01, determined by Fisher’s exact test. The numbers of neurons are indicated above the bars. For the RNAi experiment, Rtnl2 data is shown as the matched control and yw (no RNAi hairpin) control data was used for the overexpression comparison. (G) Dendrite injury was performed in Drp1 RNAi neurons without pre-axotomy. Dendrite degeneration assayed at the indicated times. Control data from Fig 1E is included for comparison. The numbers of cells analyzed for each condition are shown above the bars.

To understand how mitochondria might regulate axotomy-induced NP, we compared mitochondrial shape in dendrites before and 8h post axon injury (hpa). Mitochondria were labeled with mito-GFP and membranes with mCD8-RFP. The average length of mitochondria decreased significantly after injury (Fig 2A and 2B). Specifically, more short (magenta arrows Fig 2A) and fewer long mitochondria (orange arrows, Fig 2A) were present at 8hpa (Fig 2C). The total number of mitochondria in dendrites also increased at 8hpa (Fig 2D).

To determine whether mitochondrial fission was responsible for the changes in mitochondria length after axon injury, we used RNAi to target Drp1, a dynamin-related GTPase that mediates mitochondrial fission [23]. Drp1 RNAi hairpins were expressed in ddaE neurons under control of 221-Gal4, together with Dicer2, mito-GFP and mCD8-RFP. One of the Drp1 RNAis (referred to as Drp1 RNAi #2) dramatically elongated mitochondria in dendrites and led to clustering of mitochondria in the cell body (S1A Fig). Injury-induced changes in mitochondria were not visible in this background (S1A Fig). However, mitochondrial shape was so different in these cells that we looked for an alternate Drp1 RNAi line that would not have such a strong effect in uninjured neurons. In neurons expressing a different Drp1 RNAi, mitochondrial length was fairly normal in uninjured cells (S1B Fig). The only significant difference was a decrease in the number of mitochondria under 0.5 μm, which is consistent with partial reduction of Drp1 protein levels. Although the effects in uninjured cells were subtle, this Drp1 RNAi also completely eliminated the axotomy-induced changes in length and number (Fig 2A’–2D’). Because this RNAi line eliminated injury-induced mitochondrial fission without dramatically altering baseline mitochondrial length we used it for the subsequent experiments. Mitochondrial motility was also upregulated in dendrites after axon injury, but this change was not related to Drp1-mediated fission (S1C Fig).

To determine whether the increase in mitochondrial fission induced by axon injury was related to downregulation of NP by mitochondria, we assayed NP in Drp1 RNAi neurons. As in Miro and milton RNAi neurons, Drp1 RNAi increased the level of protection, while overexpression of Drp1 had the opposite effect (Fig 2E and 2F). Drp1 RNAi did not influence the normal time course of dendrite degeneration (Fig 2G), thus Drp1 only affects degeneration after axotomy in the NP assay, but has no effect on the baseline rate of dendrite degeneration in the absence of axon injury.

### Caspase reduction increases axotomy-induced neuroprotection

Drp1-mediated mitochondrial fission occurs during apoptosis in *Drosophila* and other organisms [24, 25], and mitochondria and caspases have been linked in a neurodegenerative response triggered by glial signaling [26]. In mammals and in *C*. *elegans* fission is upstream of caspase activation [27, 28]. Because of this connection between mitochondrial fission and caspase activation, we hypothesized that caspases might also inhibit axotomy-induced NP.

**Fig 3:**
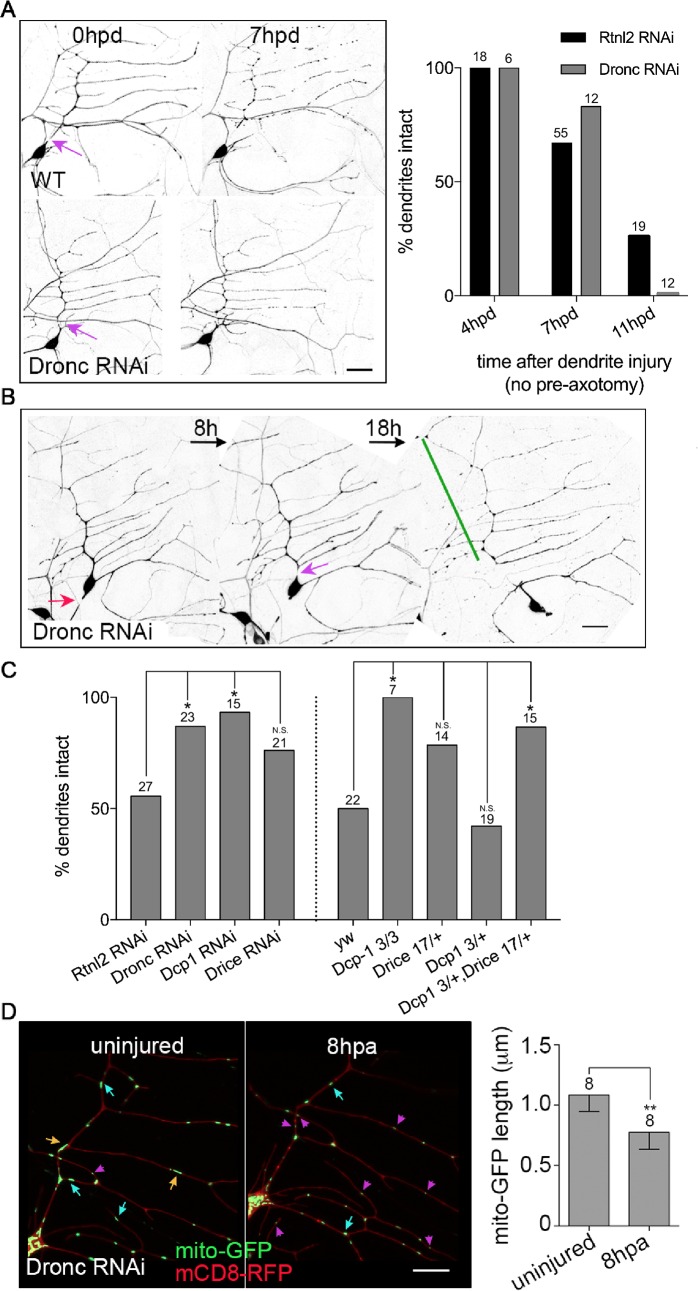
Caspases inhibit axotomy-induced neuroprotection. (A) Left, images of control and Dronc RNAi neurons in which dendrites were severed without axon pre-cut are shown. Neurons were labeled with EB1-GFP. Purple arrows indicate the site of dendrite injury. The scale bar is 20 μm. Right, presence of intact dendrites was scored at different time points. Control data from Fig 1E is included for comparison. The numbers of neurons analyzed for each condition are shown above the bars. (B) The NP assay was performed in Dronc RNAi neurons labeled with EB1-GFP. Red arrows indicate site of axon injury; purple arrows, dendrite injury; green lines, stabilized dendrites. The scale bar is 20 μm. (C) Quantification of NP is shown. The numbers on the bars indicate the numbers of neurons analyzed. Control data (Rtnl2 for RNAi and yw for other genotypes) from Figs 1D and 2F is shown for comparison. A Fisher’s exact test was used to determine statistical significance. * p<0.05. N.S. not significant. (D) Left, images of mitochondria in the dendrites of Dronc RNAi neurons before and 8h post axon injury are shown. Orange, blue and magenta arrows indicate long, medium and short mitochondria respectively. The scale bar is 10 μm. Right, quantification of mito-GFP length is shown. The numbers of neurons analyzed are indicated above the bars. ** p<0.01, determined with a t test. Error bars are SD.

To test this hypothesis we expressed large RNA hairpins to target the initiator caspase Dronc and assayed both dendrite degeneration and NP. Dendrite degeneration is normally complete by 18h after severing, and blocking caspases does not alter this [22]. To test whether caspase reduction might subtly alter the timing of dendrite degeneration, we assayed earlier timepoints, and again found that the dendrite degeneration proceeded was not influenced by caspase reduction (Fig 3A). However, Dronc RNAi did result in a significantly higher level of axotomy-induced NP compared to control cells (Fig 3B and 3C) consistent with a role for Dronc in negative regulation of NP.

We also tested whether Dcp-1 and Drice, two effector caspases, inhibit axotomy-induced NP. Indeed, both RNAi and a strong loss-of-function mutant of Dcp-1, *Dcp-1*^*3*^ [29], increased NP (Fig 3C). Drice RNAi and heterozygous *Drice*^*17*^ [30] neurons also had higher NP than control, but the results were not significantly different. It was not possible to test homozygous *Drice*^*17*^ mutants as these animals die, so we introduced one copy of *Drice*^*17*^, into heterozygous *Dcp-1*^*3*^ mutant animals, and this significantly enhanced protection (Fig 3C), indicating both effector caspases are likely to be involved in negative regulation of axon injury-induced protection.

Although in *C*. *elegans* and mammals, mitochondrial fission and Drp1 act upstream of caspases [27, 28], in *Drosophila* the effector caspase Dcp-1 can regulate mitochondrial shape and function [31]. To determine whether caspases were required for injury-induced mitochondrial fission, we assayed fission in Dronc RNAi neurons. Mitochondrial length still decreased in response to axon injury in Dronc RNAi neurons (Fig 3D), suggesting caspases do not act upstream of mitochondrial fission, but may be downstream as in other organisms. However, we cannot rule out that caspases and Drp1 act independently to dampen NP.

### Nmnat is required for axotomy-induced neuroprotection

**Fig 4:**
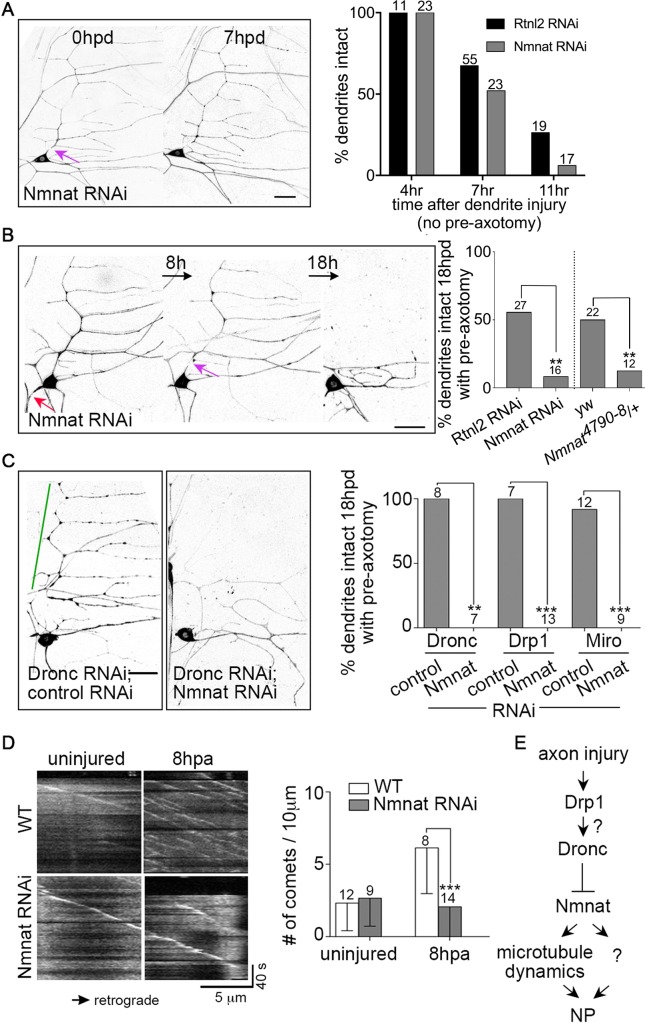
Nmnat is required for stabilization of dendrites in response to axotomy. (A) Left, dendrites of Nmnat RNAi neurons were severed without axon pre-cut. Neurons were labeled with EB1-GFP. The scale bar is 20 μm. A purple arrow marks the site of dendrite injury. Right, quantification of dendrite degeneration at various time points is shown with control data from Fig 1E for comparison. * The numbers above the bars are the number of cells analyzed for each condition. (B) Left, the NP assay was performed in Nmnat RNAi neurons labeled with EB1-GFP. A red arrows shows the site of axon injury, a purple arrow, dendrite injury. The scale bar is 20 μm. Right, quantification of NP is shown, with control data from Figs 1D and 2F. The numbers of neurons analyzed are indicated on the bars. ** p<0.01, determined by Fisher’s exact test. (C) The NP assay was performed in neurons expressing Dronc RNAi in conjunction with a control RNAi or Nmnat RNAi in cells labeled with EB1-GFP. Images of neurons 18hpd are shown. The green line indicates a stabilized dendrite. The scale bar is 20 μm. Right, quantification of NP is shown. The numbers of neurons analyzed are indicated above the bars. A Fisher’s exact test was used to determine statistical significance. ** p<0.01, *** p<0.001. (D) Left, kymographs of EB1-GFP in the dendrites of control and Nmnat RNAi neurons before and 8h after axon injury are shown; the cell body is off to the right in each image. The trajectory of EB1 comets appears white. The X- and Y- axes represent distance and time, respectively. Right, microtubule dynamics is quantified. The numbers of neurons analyzed are indicated above the bars. *** p<0.001, determined by unpaired t test. Error bars are SD. (E) A proposed model that summarizes the finding so far is shown. Steps that cannot be definitely resolved with the data are indicated by question marks.

Thus far we have identified mitochondrial fission and caspases as negative regulators of NP. We also wished to identify positive regulators. As Nmnat, a conserved NAD+ biosynthetic enzyme, can protect neurons from degeneration induced by long poly-Q proteins [32] and tau [33], we tested whether it might also be involved in injury-induced NP. Indeed, Nmnat RNAi completely eliminated NP induced by axon injury (Fig 4B). To test the specificity of this effect, we also assayed dendrite degeneration without prior axon injury. We did not find any changes in the timing of degeneration in the absence of prior axon injury (Fig 4A) despite previous association of Nmnat with dendrite stability. The previous studies were done in class IV neurons, which have much larger and more complex dendrite arbors than the neurons used here, and exhibit gradual loss of complexity over time in Nmnat heterozygotes [34]. We did not observe any differences in arbor structure in ddaE neurons, and the previous degeneration was observed over days rather than hours.

To confirm a role for Nmnat in injury-induced degeneration, we assayed NP in both Nmnat heterozygous mutant animals (Fig 4B). The mutant is a previously characterized null allele of *Nmnat* [35]. NP was eliminated in this genetic background (Fig 4B), suggesting that normal levels of Nmnat are required for NP. As the phenotype in the Nmnat RNAi and heterozygous mutant animals was similar, we used an antibody to Nmnat to stain Nmnat RNAi neurons. We observed about 50% reduction in Nmnat signal in these neurons compared to control (S2A Fig), consistent with partial reduction of Nmnat protein in the RNAi experiment.

To determine whether the elimination of NP in animals with a partial reduction of Nmnat were due to a general inability to respond to injury, we tested whether Nmnat RNAi neurons could regenerate axons. When ddaE neurons are axotomized close to the cell body, axon regeneration proceeds by converting a dendrite into a growing axon [36]. Nmnat RNAi neurons were fully capable growing a new axon from a dendrite after a proximal axotomy, indicating the cell can mount at least one demanding injury response (S2B Fig). Together, these results suggest that a partial knockdown of Nmnat in ddaE neurons does not alter the ability of the cell to sense and respond to axon injury. Therefore the loss of NP in this background is most likely due to a specific role of Nmnat in injury-induced protection.

We next tested how Nmnat reduction impacts the increased stabilization that occurs when Miro, Drp1 and Dronc are reduced. We found that Nmnat RNAi completely eliminated the increase in NP caused by Miro, Drp1 and Dronc RNAi (Fig 4C). This result suggests that negative regulation of NP by caspases acts upstream of Nmnat.

To try to position Nmnat relative to other regulators of NP, we examined microtubule dynamics. We previously found that microtubule dynamics, specifically the number of growing plus ends, is dramatically upregulated in dendrites after axon injury in sensory neurons [29], and that this increase in dynamics acts to stabilize dendrites against degeneration [8]. To test whether microtubules and Nmnat protect dendrites in the same pathway or parallel pathways, we labeled the growing ends of microtubules using EB1-GFP in ddaE neurons. We then compared microtubule dynamics in control and Nmnat RNAi neurons. Nmnat reduction specifically abolished the increase in microtubule dynamics at 8hpa without influencing the base-line microtubule dynamics (Fig 4D). Thus the upregulation of microtubule dynamics after axon injury requires Nmnat. It seems unlikely, however, that microtubule dynamics is the sole effector of Nmnat as dampening microtubule dynamics does not block injury-induced protection as strongly or consistently as reducing Nmnat (Fig 4B and 4C and [8]). In summary, our results lead to a model in which Nmnat is a central effector of NP acting upstream of microtubule dynamics and downstream of negative regulation by Drp1 and Dronc (Fig 4E).

### Nmnat overexpression is sufficient to stabilize dendrites and increase microtubule dynamics

**Fig 5:**
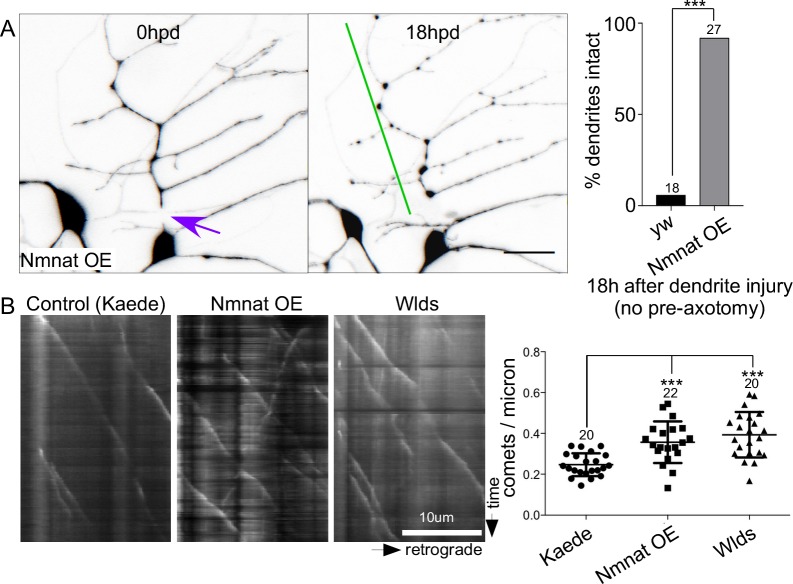
Nmnat is sufficient to delay dendrite degeneration and increase microtubule dynamics. (A) Dendrites were severed in neurons expressing GFP-Nmnat-B-delta N without prior axotomy. An example of a cell immediately after dendrite injury and then 18h later is shown with the injury site indicated by a purple arrow and persistent dendrite with a green line. The scale bar is 20 μm. Quantitation of dendrite degeneration is shown at the right. In control (yw) and Nmnat overexpressing neurons, the number of intact dendrites was scored 18h after dendrites were severed. A Fisher’s exact test was used to calculate significance with *** indicating p<0.001. The number of cells analyzed for each genotype is shown above the bars. (B) Movies of EB1-GFP were acquired in the trunk of the ddaE comb dendrite. Neurons expressed a control protein, Kaede, GFP-Nmnat-B-deltaN or Wlds. Kymographs from a portion of the dendrite are shown with the cell body to the right. Quantitation of comet number in the different genetic backgrounds is shown at the right. The central line shows the mean and the error bars are the SD. Numbers of cells analyzed are shown above the plots. Significance was calculated with an unpaired t test and * indicates p<0.05.

As Nmnat seemed so closely linked to NP and was required for both stabilization and increased microtubule dynamics induced by axon injury, we tested whether it was sufficient to induce these responses. We therefore expressed GFP-tagged Nmnat-B-delta-N. The delta-N refers only to a difference from the cDNA used to the annotated cDNA in flybase (see next section in results). In the background of Nmnat-B overexpression we severed a dendrite without prior axon injury and scored its presence 18h later. In control neurons almost no dendrites remained at this time, while in the Nmnat-expressing neurons almost all were intact (Fig 5A). Because Nmnat was sufficient to protect dendrites in the absence of axon injury, we also tested whether it was sufficient to increase microtubule dynamics in uninjured neurons. As a control we expressed a soluble fluorescent protein, Kaede. Expression of either GFP-Nmnat-B-delta-N and Wlds (mouse Nmnat1 with an additional stretch of amino acids at the N-terminus) increased the number of growing microtubule ends in dendrites of uninjured neurons (Fig 5B). Thus Nmnat is not only required for injury-induced NP, but is sufficient both for NP and the associated increase in microtubule dynamics.

### Dronc tunes Nmnat-mediated neuroprotection to promote regeneration

While increased stability is likely to help neurons to survive after axon damage, the reason for limiting stability through caspase activity is not intuitive. However, the timing of events triggered by axon injury suggested a hypothesis. Axotomy-induced NP is maximal 8-24h after axon injury in ddaE neurons (Fig 1A and 1A’ and [8]), while axon regeneration typically begins 24-48h after injury in these cells [36]. We therefore hypothesized that turning down early NP might promote subsequent regeneration.

**Fig 6:**
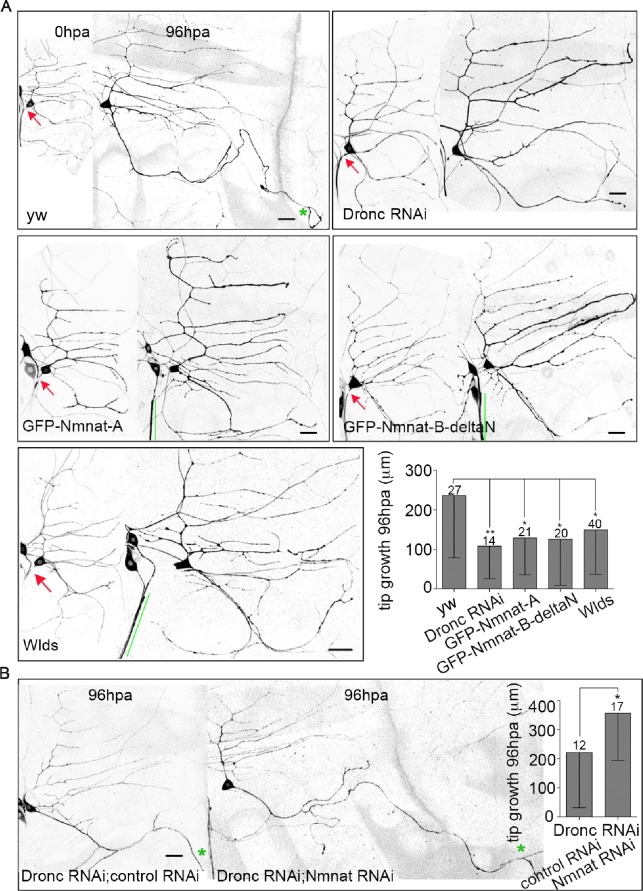
Excess Nmnat suppresses axon regeneration. (A) Images of neurons of the indicated genotypes 0h and 96h after proximal axotomy are shown. The neurons are labeled with EB1-GFP. Red arrows indicate the site of axon injury. A green star marks the tip of the dendrite that converts to an axon after injury; all neurites that grew more than 50 microns have a star. Green lines mark cut off axons that have not degenerated. Scale bars are 20 μm. The average amount of tip growth (growth beyond the length increase due to growth of the whole animal) of the regenerating axon 96h after injury is shown in the graph. The numbers of neurons analyzed are indicated above the bars. Error bars, SD. See methods for statistics. Error bars represent SD. * p<0.05, ** p<0.01. (B) Axon regeneration of ddaE neurons co-expressing Dronc RNAi with a control RNAi or Nmnat RNAi was assayed. Green stars mark tips of the converted dendrites. The scale bar is 20 μm. Statistical significance was determined with an unpaired t test. * p<0.05. Error bar show SD. The numbers of neurons analyzed are indicated above the bars.

To test whether uncontrolled NP might inhibit regeneration, we compared regeneration in control and Dronc RNAi neurons. In control neurons the average amount of new axon growth 96h after injury was over 200 microns, but in Dronc RNAi neurons the average growth was less than half of that (Fig 6A). Dronc activity therefore promotes regeneration, perhaps by limiting axotomy-induced NP.

If reduced regeneration in Dronc RNAi neurons is due to overactive Nmnat, we predict that Nmnat overexpression should lead to a similar defect in axon regeneration. To test this idea, we generated transgenic flies that encode GFP-tagged *Drosophila* Nmnat. Two splice forms of Nmnat exist in *Drosophila*. Nmnat-A contains a nuclear localization signal (NLS) while Nmnat-B does not. The Nmnat-B described in flybase has 31 amino acids at its N-terminus that are not encoded in any existing cDNAs; our GFP-Nmnat-B does not have these 31 amino acids, so we refer to it as Nmnat-B-deltaN.

Consistent with our hypothesis, over-expression of either Nmnat isoform suppressed axon regeneration (Fig 6A). In addition, over-expression of Wlds [37], which includes mouse Nmnat1 and 70 additional amino acids [38], had the same effect (Fig 6A). HA-tagged Nmnat [35] had a similar, although not statistically significant, effect (S3A Fig). These results are consistent with previous studies showing that Wlds overexpression can lead to reduced axon regeneration in a variety of cell types and contexts [39–42].

To determine whether Nmnat might act to dampen regeneration downstream of Dronc, we paired Dronc RNAi with Nmnat RNAi to see if reducing Nmnat would rescue the Dronc RNAi phenotype. To control for potential Gal4 dilution effect when expressing many UAS-driven transgenes together, we paired Dronc RNAi with a control RNAi. Indeed, the addition of the control transgene reduced the effect of Dronc RNAi on regeneration (Fig 6). However, in Dronc plus Nmnat double RNAi neurons, regeneration was significantly enhanced compared to the matched control (Fig 6B). This result is consistent with Nmnat acting as a negative regulator of regeneration downstream of Dronc. Levels of regeneration in this experiment were higher than in other genetic backgrounds. It is possible that Dronc also targets positive regulators of regeneration that can increase outgrowth when Nmnat-mediated in inhibition of regeneration is reduced.

Although we found Nmnat was central to NP, we did not see large changes in amount or distribution of endogenous Nmnat in ddaE neurons after injury using immunofluorescence (S3D Fig). This may be because small or transient changes in levels or activity of Nmnat are sufficient to stabilize dendrites, and because endogenous Nmnat was difficult to detect. GFP-Nmnat-B-deltaN was evenly distributed in the nucleus and cytoplasm and did not change its localization in response to injury (S3B Fig). In uninjured ddaE neurons, GFP-Nmnat-A was detected primarily in nuclei (S3C Fig). At 8h post axon injury, the ratio of nuclear to cytoplasmic Nmnat-A signal was significantly decreased (S3C Fig). In contrast, the ratio did not change in response to axotomy in Dronc RNAi neurons (S3C Fig). Although we do not know the significance of the decrease in nuclear Nmnat-A relative to cytoplasmic, the fact that it is dependent on Dronc is consistent with Dronc regulating Nmnat after axon injury.

There are two ways excessive Nmnat could dampen regeneration: either by generating a persistent stump that blocks regeneration or more directly from within the regenerating cell. A persistent axon stump could block or repel new axon growth, as has been demonstrated in zebrafish [42]. In the regeneration assay used here, physical block by the stump cannot be important as the new axon grows from a dendrite on the opposite side of the cell. To test whether a persistent stump might influence regeneration in some other way, we expressed the Wlds protein in ddaC neurons, which are next to ddaE neurons. This approach enabled generation of a persistent stump near a cell body that did not itself express extra Wlds or Nmnat. When we severed axons of both the Wlds-expressing cell (ddaC) and wild-type ddaE, the ddaC axon persisted as expected, and regrowth of the axon from a dendrite occurred normally in the ddaE neuron (S3E Fig). Failure of a neighboring persistent stump to reduce regeneration is consistent with excessive Wlds or Nmnat acting cell-autonomously to dampen regeneration.

### Positive and negative regulation of NP occurs downstream of conserved axon injury signals

**Fig 7:**
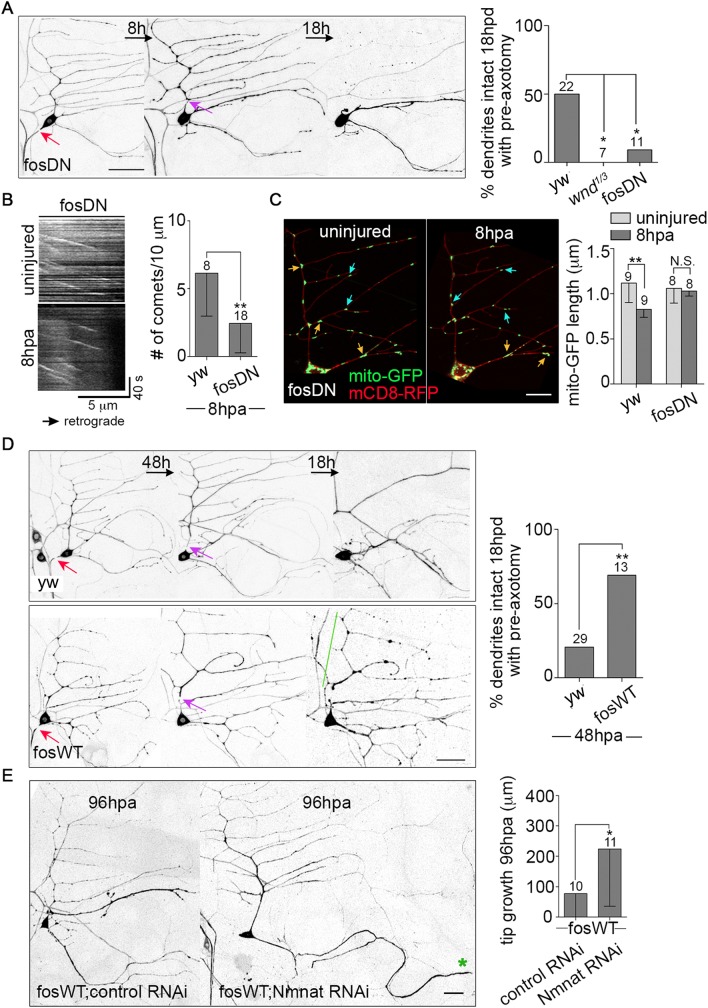
DLK/fos signaling coordinates neuroprotection and axon regeneration. (A) Left, the NP assay was performed in fosDN-expressing neurons labeled with EB1-GFP. Red arrows point to the site of axon injury, purple arrows, dendrite injury. The scale bar is 50 μm. Right, quantification of NP is shown. The numbers of neurons analyzed are indicated above the bars, and control data is from Fig 2F. Statistical significance was determined with a Fisher’s exact test. * p<0.05. (B) Left, kymographs of EB1-GFP comets in fosDN-expressing neurons before and 8h after axon injury are shown. The graph at the right shows the quantification of microtubule dynamics in the ddaE comb dendrite 8h after axon injury. The numbers of neurons analyzed are indicated above the bars; control data is from Fig 4D. ** p<0.01, determined with an unpaired t test. Error bars are SD. (C) Left, mitochondria were imaged in the dendrites of fosDN-expressing neurons before and 8h post axon injury. mito-GFP (mitochondria) and mCD8-RFP (cell membrane) were expressed under the control of 221-Gal4. Orange and blue arrows indicate long (>1.5 μm) and medium (1–1.5 μm) mitochondria, respectively. The scale bar is 10 μm. Right, quantification of the average length of mitochondria in fosDN neurons is shown. Control data from Fig 2B is included for comparison. The numbers of neurons analyzed are indicated above the bars. A t test was used to test for significance. Error bars are SD. (D) Left, the NP assay was performed in WT and fos-overexpressing (fosWT) neurons as in (A), except that 48h elapsed between axon injury and dendrite injury rather than the 8h used elsewhere. Red arrows indicate the site of axon injury and purple arrows, dendrite injury. The green line marks a stabilized dendrite. The scale bar is 50 μm. Right, quantification of NP with the 48h gap between axon and dendrite injury is shown. The numbers of neurons analyzed are indicated above the bars. ** p<0.01, determined by Fisher’s exact test. (E) Left, images of neurons that co-express fosWT with a control RNAi or Nmnat RNAi at 96h post axon injury are shown. The green star marks the tip of the converted dendrite. The scale bar is 20 μm. Right, the average amount of regeneration was quantified and shown in the graph. The numbers of neurons analyzed were indicated above the bars. * p<0.05, determined by an unpaired t test. Error bars are SD.

Thus far we have shown that Nmnat is required for NP, that caspases limit NP, and that overactivation of NP dampens regeneration. However, there must also be positive signals that turn NP on in response to axon injury. Indeed, we previously showed that JNK is required for NP mediated by increased microtubule dynamics [8]. JNK can act downstream of DLK in initiation of axon regeneration in an injury-induced cascade that results in fos-mediated transcription in Drosophila [10]. We therefore tested whether DLK and fos played a role in NP. A trans-heterozygous combination of *wnd* alleles (wnd is the name for Drosophila DLK), and a fos dominant negative (fosDN) transgene have been shown to block injury signaling [10] and regeneration [43], so we used these tools to test for a role in NP. In both genetic backgrounds induction of NP by axon injury was completely blocked (Fig 7A). In addition, fosDN blocked the increased microtubule dynamics in dendrites after axon injury (Fig 7B). We conclude that the DLK/JNK/fos pathway is required for NP and its associated upregulation of microtubule dynamics. The NP that protects dendrites in sensory neurons may therefore be similar to the DLK and fos-mediated axon stabilization induced by crushing motor axons [7].

We also tested whether the fos pathway might be upstream of mitochondrial fission induced by axon injury. Unlike control neurons (Fig 2A and 2B), no decrease in mitochondrial length was observed in fosDN neurons (Fig 7C). Thus fos activity is required for injury-induced mitochondrial fission. This suggests fos is required both to turn on NP and to induce mitochondrial fission that limits NP.

**Fig 8:**
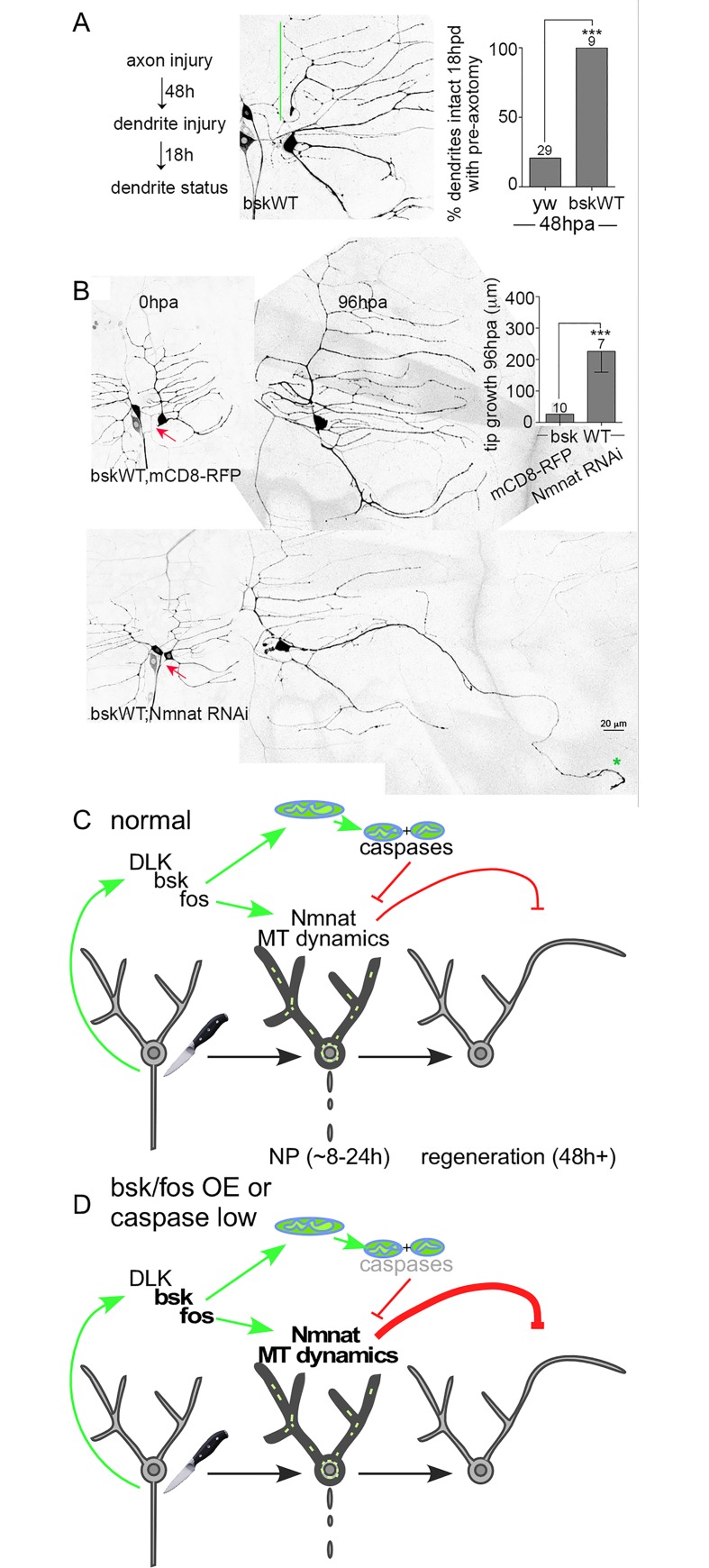
Effects of JNK (bsk) overexpression on NP and axon regeneration, and a summary model. (A) A 48h NP assay was performed in bsk-overexpressing neurons. The green line indicates a stabilized dendrite. Statistical significance was determined by a Fisher’s exact test and the numbers above the bars indicate numbers of neurons analyzed. *** p<0.001. Control data is from Fig 7D. (B) Axon regeneration assays were performed in neurons overexpressing bsk paired with either mCD8-RFP or Nmnat RNAi. UAS-mCD8-RFP was expressed as a control for Nmnat RNAi to keep the number of UAS-controlled transgenes constant and rule out Gal4 dilution effects. Red arrows mark sites of axon injury. The green star indicates the tip of the converted dendrite. Statistical significance was determined by a Mann-Whitney test. Error bars represent SD. *** p<0.001. (C) A summary model of the results is shown. Axon injury activates the DLK/bsk/fos response pathway. The AP-1 transcription factor fos turns on early injury responses that include Nmnat-mediated NP (indicated by darker cell outline in middle image), microtubule dynamics (short green lines in middle image) and mitochondrial fission. NP is mediated by Nmnat, which, if unchecked dampens subsequent regeneration. Caspases and mitochondrial fission counteract NP. (D) Reduction of caspases, increased bsk or fos, or increased Nmnat result in excess or longer than normal NP. Unbalanced NP dampens regeneration.

If fos is a critical regulator of NP, then its overexpression might alter the time course of dendrite stabilization. Indeed, in control neurons NP is low 48h after axon severing, but in fos overexpressing neurons it remained high (Fig 7D). This is consistent with previous studies showing that fos can stabilize axons in other situations [7, 44]. Overexpressing fos also blocked axon regeneration (Fig 7E). To determine whether the inhibition of regeneration by fos was due to excessive NP, we co-expressed Nmnat RNAi with fos. As in other experiments with multiple transgenes we paired fos with a control RNAi so that transgene number was matched. Nmnat RNAi completely rescued regeneration in the fos overexpression (Fig 7E). We conducted similar experiments with overexpressed bsk, the JNK homolog in *Drosophila*. Like fos, bsk overexpression extended protection (Fig 8A), and blocked regeneration in a Nmnat-dependent manner (Fig 8B). Together these results demonstrate that overexpression of fos or JNK extends the normal timing of NP and, in a Nmnat-dependent manner, reduces regeneration. Thus Nmnat is a both a positive regulator of NP and a negative regulator of regeneration that can act downstream of JNK and fos signaling.

## Discussion

Our results lead to a model (Fig 8C) in which axon injury triggers opposing responses downstream of the initial DLK/JNK/fos signaling cascade. One early output of this conserved injury response pathway is NP, a global stabilization of the parts of the neuron still connected to the cell body. The central mediator of NP is Nmnat. One Nmnat effector is the dramatic increase in microtubule dynamics observed after axon injury. As axon damage is likely to be accompanied by disturbances in the surrounding tissue, making the cell more resistant to degeneration by turning on NP may help the neuron survive the initial trauma.

Fos injury signaling also triggers Drp1-mediated mitochondrial fission in the first few hours after axon injury, and this leads to dampening of NP by caspases. We envision positive and negative regulation of NP balancing one another in different ways through time after injury. Eventually the negative pathway must outweigh the positive or regeneration is dampened by persistent NP (Fig 8D). It is possible that the timing of this balance shift is controlled by additional signals that report whether the environment is conducive for regeneration.

This model suggests that rather than DLK/JNK/fos directly regulating regeneration, this signaling pathway kicks off a multi-step response to axon injury that includes regeneration as a relatively late event. Indeed, although this pathway is known as the conserved axon regeneration pathway, we find that it first turns on a response that inhibits regeneration. Although this idea is surprising, this model does make sense in the overall picture of neuronal injury responses and stabilization. For example, in mammals [45, 46] and flies [10] the AP-1 transcription factor fos is activated soon after axon injury, but its role in regeneration is not as clear as that of some other transcription factors like jun. Our data suggests that this early activation could be because fos orchestrates the injury response that precedes regeneration.

Our results also touch on the role of caspases in axon regeneration. A study in *C*. *elegans* demonstrated that caspases are positive regulators of axon regeneration [47], which is surprising considering their involvement in self-destruct programs like apoptosis and dendrite pruning. We confirm that in *Drosophila* caspases are pro-regenerative. In addition, our data suggests that this effect is not through a direct role in regeneration, but because caspases down-regulate NP, which inhibits regeneration.

A negative role for mitochondria in NP is also intriguing. Mitochondria seem to promote axonal stability [15], and there are studies in several systems that suggest the neuroprotective effects of Nmnat or Wlds require mitochondria [17–19]. However, mitochondria can play prodegenerative roles in other contexts [26, 48]. More specifically mitochondrial fission can promote degeneration [49]. Here we demonstrate that mitochondria, Drp1 and caspases all counteract NP, suggesting that caspase activation may regulate NP downstream of mitochondrial fission. This does not mean that mitochondria are not also positive regulators of this type of NP. Indeed the data in this study combined with others suggests that mitochondria are critical nodes for control of neuronal stability and both positive and negative regulation likely converge on them.

Like mitochondria, the role of Nmnat in injury responses has been difficult to classify simply as either positive or negative. Its ability to prevent injury-induced Wallerian degeneration, as well as to act as an endogenous neuroprotective factor [50] has led to the idea that it has a purely positive influence on neuronal health. However, the myriad ways in which it can be regulated [50] suggest that it is useful only in exactly the right dose. Indeed we show that when its regulation is disrupted, Nmnat inhibits a different type of neuronal resilience: axon regeneration. Thus upregulation of Nmnat as a potential therapeutic strategy to counteract neurodegeneration could have negative outcomes due to dampened regeneration.

While our experiments support the idea that endogenous Nmnat is a central regulator of neuronal stability, the way it exerts this effect remains unclear. Nmnat is an enzyme that uses ATP and NMN (nicotinamide mononucleotide) to make NAD+. Protective effects of endogenous or overexpressed Nmnat have been proposed to be due to maintenance of high NAD levels [51–53], keeping levels of the precursor NMN low [54], acting as a chaperone [32, 35], and through maintaining mitochondrial integrity or function [17–19]. We now show that Nmnat also acts upstream of increased microtubule dynamics after axon injury. This Increased microtubule dynamics in response to axon injury is also seen in mammalian neurons, and so this part of the NP response is likely to be conserved [13]. Although we have previously shown increased microtubule dynamics plays a role in NP [8], and now show that Nmnat overexpression is sufficient to increase microtubule dynamics, it is possible that Nmnat has other effectors that can mediate NP.

In conclusion, we propose a model in which DLK signaling initiates key injury responses before axon regeneration begins. These responses include upregulation of Nmnat-mediated NP, microtubule dynamics and mitochondrial fission. Mitochondrial fission likely counteracts NP through caspase activation, although it is possible that mitochondria and caspases regulate NP independently. Although this early response is downstream of the core axon regeneration kinase cascade, it actually inhibits regeneration if unchecked. This multi-step model of injury responses downstream of DLK helps explain the function of caspases in promoting regeneration. We anticipate that understanding the transition between early injury responses and regeneration itself will suggest strategies for promoting axon regeneration without overactivating NP, which would, in turn, dampen regeneration. A more complete understanding of the relationship between NP and regeneration is essential to designing any therapeutic approach to either stabilize neurons or to enhance regeneration.

## Materials and Methods

### *Drosophila* stocks

The following RNAi fly strains were used in this study: Rtnl2 (33320) [8], gammaTub37C (25271) [8], Miro (106683) [22], milton (41508) [55], Dronc (23035) [22], Dcp-1 (107560) Drice (28065) [56] and Drp1 (44156, referred to as #2) from the Vienna Drosophila RNAi Center, and Drp1 (27682), Nmnat (29402) [18] from the Bloomington Drosophila Stock Center (BDSC). All RNAi transgenes were coexpressed with UAS-Dcr2 to increase knockdown efficiency [57]. Other lines include 221-Gal4, ppk-Gal4, UAS-mito-GFP (BDSC 8443), UAS-EB1-GFP, UAS-mCD8-RFP, UAS-Drp1 [58], UAS-Wlds [37], *Nmnat*^*delta4790-8*^ [35], *Drice*^*17*^ [30], *Dcp-1*^*3*^ [29], UAS-fosWT (BDSC 7213), UAS-bsk-A.Y (BDSC 6407), *wnd*^*1*^, *wnd*^*3*^, and UAS-fosDN [59].

### Live imaging, injury and image processing

Fly embryos were collected at 20C overnight and aged at 25C for 2 or 3 days before imaging. Two days of aging was used for all axon regeneration assays because these extend 96h, and 3 days of aging were used for all other experiments. Larvae were mounted between a slide coated with a dry agarose patch and a coverslip, which was held in place with sticky tape. A MicroPoint pulsed UV laser (Andor Technology) was used to injure dendrites and axons of ddaE neurons expressing EB1-GFP or mCD8-RFP under the control of 221-Gal4. Confocal images were acquired using a Zeiss LSM510 with a 63X oil objective (NA1.4) right after injury. Larvae were then kept in individual food caps at 20C for the indicated time periods and were then reimaged using an Olympus FV1000 confocal microscope equipped with a 60X oil objective (NA1.42). Maximum intensity projections were generated using ImageJ software, and were aligned and processed using Adobe Photoshop software.

### Microtubule dynamics

To measure microtubule dynamics in dendrites after injury, we imaged neurons for at least 100 frames (1 frame/2s) using an Olympus FV1000 microscope with a 60X objective at zoom 3, and counted the total number of EB1-GFP comets in a 10 μm dendrite segment close to the cell body from 3 in-focus frames. Only comets moving in 3 consecutive frames were included for quantification. The reslice tool of ImageJ was used to generate kymographs with 1 pixel spacing.

In uninjured neurons, EB1-GFP-expressing neurons were imaged with a Zeiss AxioImager M2 equipped with LED illumination and an AxioCam 506 camera. A 63x 1.4 NA objective was used to acquire images every second. After image capture, analysis was performed in ImageJ. In each movie, the length of the comb dendrite of the ddaE neuron that was in focus was measured. EB1-GFP comets that passed through this region during the 300 seconds of the movie were counted and this number was divided by the length to get comets per length. The time (300s, with 1 frame per second) was the same in all movies and so was not included in the normalization.

### Mitochondrial imaging and analysis

Live imaging of mitochondria was performed by expressing UAS-mCD8-RFP and UAS-mito-GFP under the control of 221-Gal4. Images were taken on a Zeiss LSM510 at 1 frame/s using a 63x objective and 2x zoom. Injury-induced mitochondrial behavior changes were analyzed in dendrites in a 66.8 μm^2^ region close to the cell body. The template matching plugin in ImageJ was used to minimize the effect of larval body movements. Mitochondrial length was measured along the longest dimension of mito-GFP shapes using the measure tool in ImageJ. The average length of mitochondria was calculated from at least 8 neurons, each of which contained 13–52 mitochondria in the imaging region. To further analyze changes in length, mitochondria were grouped according to the length of mito-GFP, and the percentage of each group was calculated before and after injury.

### Axon regeneration

ddaE neurons expressing EB1-GFP were axotomized close to the cell body and reimaged after 96h. One dendrite usually extends and converts into a new axon by 96h in response to a proximal axon injury [36]. using the NeuronJ plugin in the ImageJ software, we measured the length of the specified dendrite at 0h (R0h) and 96h (R96h), and a nearby non-regenerating dendrite (NR0h and NR96h) so that normal dendrite expansion as the larva grows could be taken into account. The formula R96h-R0h*NR96h/NR0h was used to calculate growth of the new axon tip.

### Dendrite degeneration

The comb dendrite of ddaE neurons was severed close to the cell body. Degeneration speed was measured by scoring morphology of the severed dendrite at 4, 7, and 11. Dendrites with no discontinuities were scored as intact, and any breaks resulted in them being scored as not intact.

### Axotomy-induced neuroprotection (NP) assay

ddaE neurons expressing EB1-GFP were axotomized close to the cell body 8h or 48h before the dorsal comb-like dendrite was severed. Dendrite status was determined 18h post dendrite injury. In all the neurons we examined, dendrites either completely degenerate and no remnants are left or remained intact. Example images of both types of result are shown in Fig 1B. Therefore, NP is measured using the percentage of neurons with intact dendrites at 18hpd.

### Quantification and statistics

GraphPad Prism 6 software was used to generate graphs and perform statistical analysis. A Fisher’s exact test was used to determine significance of neuroprotection assays and length distribution of mitochondria. Other types of data were tested for normal distribution using D’Agostino-Pearson normality test. If the data passed the normal distribution test, a t test or in Fig 6A, a one way ANOVA followed by Dunnett’s multiple comparison test, was used to determine statistical significance. Otherwise, a Mann-Whitney test was used. Details of the specific t test performed and sample size for each experiment are described in Fig legends. Data were plotted as mean ± standard deviation (SD). * p<0.05, ** p<0.01, *** p<0.001.

### Generation of GFP-Nmnat flies

The coding sequence of *Drosophila* Nmnat isoform A was amplified from a cDNA clone using forward primer 5’-CCGGAATTCATGATTGTGAAAATCAGCTGGCCCAAG-3’ and reverse primer 5’-ATATGCGGCCGCCTAAAGTTGCACTTGGGAAATC-3’.

The coding sequence of *Drosophila* Nmnat isoform B was amplified from the UAS-Nmnat.HA construct [35] using forward primer: 5’-CCGGAATTCATGTCAGCATTCATCGAGGAAAC-3’ and reverse primer: ATATGCGGCCGCTCAAGAGTCGCATTCGGTCGGAG.

Both forward primers contain an EcoRI site and reverse primers contain a NotI site. The amplified sequences were cloned into a pUAST-GFP vector and the resulting constructs were injected into fly embryos to generate several transgenic flies. UAS-GFP-Nmnat-A4 and UAS-GFP-Nmnat-B-deltaN8 lines were used in this study.

## Supporting Information

S1 Fig Characterizing mitochondrial length and motility in dendrites after axon injury. (A) Left, images of mitochondria in Drp1 RNAi #2-expressing neurons before and 8h post axon injury. mito-GFP and mCD8-RFP mark mitochondria and the cell membrane, respectively. Right, quantification of the average length and number of mitos in Drp1 RNAi #2 neurons is shown. The numbers of neurons analyzed are indicated above the bars. Statistical significance was determined with a t test. Error bars are SD. (B) The length distribution, average length and number of mitochondria are compared between WT and Drp1 (#1) RNAi neurons. Data of uninjured neurons from Fig 2B–2D’ were regraphed here to give a side-by-side comparison. (C) Left, kymographs of mito-GFP in the dendrites of wide-type neurons before and 8h post axon injury are shown. The X- and Y-axes represent distance and time, respectively. Right, quantification of mitochondrial motility is graphed. The numbers on the graph bars are the total numbers of mitochondria analyzed. Data were obtained from over six neurons for each genotype. Statistical significance was determined with a Fisher’s exact test. * p<0.05, ** p<0.01.
(JPG)

S2 Fig Phenotypes of Nmnat RNAi neurons. (A) Left, WT larvae and larvae in which Nmnat RNAi was expressed in several sensory neurons under control of 221-Gal4 were fileted, fixed and immunostained for endogenous Nmnat; for more information see S1 Methods. EB1-GFP was used as a cell marker. The squares outline ddaE cell bodies. Right, quantification of Nmnat intensity is shown. n = 8 neurons from 3 fillets. ** p<0.01, determined by an unpaired t test. Error bars are SD. (B) Axon regeneration was assayed in Nmnat RNAi neurons after proximal axotomy. Neurons were labeled with EB1-GFP driven by 221-Gal4. The red arrow shows the site where the axon was cut. The green stars indicate the tips of the dendrite that was converted into a regenerating axon after injury. The numbers of neurons analyzed are indicated above the bars. Statistical significance was determined by an unpaired t test. Error bars represent SD. (C) Axon degeneration was assayed in neurons expressing a control RNAi (Rtnl2) or Nmnat RNAi. Axons were severed at 0h, and then their integrity was assayed 6h and 12h later. Statistical significance was calculated with a Fisher’s exact test and * indicates p<0.05.
(JPG)

S3 Fig Nmnat localization in uninjured and injured neurons. (A) Left, the axon regeneration assay was performed in UAS-HA-Nmnat and EB1-GFP-expressing neurons. The red arrow indicates the site of axon injury. The green stars indicate the tips of the dendrite that was converted to an axon at 96hpa. Right, quantification of the average length of regeneration is shown. The numbers of neurons analyzed are indicated above the bars. An unpaired t test was used to determine statistical significance. Error bars represent SD. (B) Left, GFP-Nmnat-B-deltaN was expressed in ddaE neurons under the control of 221-Gal4. The ratio of fluorescence intensity in the nucleus and cytoplasm was compared right after axotomy (0hpa) and 8hpa; see S1 Methods. The numbers of neurons analyzed are indicated on the bars. Statistical significance was determined by a paired t test. Error bars represent SD. (C) Left, GFP-Nmnat.A in ddaE neurons was imaged 0hpa and 8hpa. mCD8-RFP was co-expressed to mark cell membrane. Middle, quantification of the nuclear/cytoplasmic ratio of GFP intensity in WT neurons is shown. Right, quantification of the nuclear/cytoplasmic ratio of GFP intensity in Dronc RNAi neurons is shown. The numbers of neurons analyzed are indicated above the bars. Statistical significance was determined by a paired t test. Error bars represent SD. * p<0.05. (D) Left, endogenous Nmnat was stained in larval fillets from animals expressing mCD8-RFP in class I neurons; the ddaE neuron is indicated with arrows. Uninjured ddaE neurons or neurons 8h after axon severing are shown. Middle, quantification of the nuclear/cytoplasmic ratio of endogenous Nmnat is shown. Right, quantification of overall Nmnat intensity in the cell body of ddaE neurons is shown. The numbers of neurons analyzed are indicated on the bars. Data were obtained from 3 to 4 fillets. An unpaired t test was used to determine if any differences were significant. Error bars, SD. (E) Left, ddaC and ddaE neurons were labeled with EB1-GFP under the control of 221-Gal4. A persistent axon stump (white line) was generated by severing the axon of ddaC neuron expressing an RFP tagged-Wlds (red nucleus) under a ppk protomer. Arrows indicate sites of axon injury. Stars mark tips of the converted dendrite. The scale bar is 20 μm. Right, quantification of axon regeneration in ddaE neurons is shown. The number of cells analyzed is shown above the bars. An unpaired t test was used to determine statistical significance. Error bars show SD.
(JPG)

S1 Methods Methods used in the Supporting Figures. Methods including Nmnat immunofluorescence, analysis of mitochondrial motility, and generation of ppk-Wlds-td flies are described.
(DOCX)
