# Supplementary figures and images for: Mitochondria and Caspases Tune Nmnat-Mediated Stabilization to Promote Axon Regeneration
Source: PLoS Genet. 2016 Dec 6;12(12):e1006503. doi: 10.1371/journal.pgen.1006503 (PMC5173288; doi:10.1371/journal.pgen.1006503)

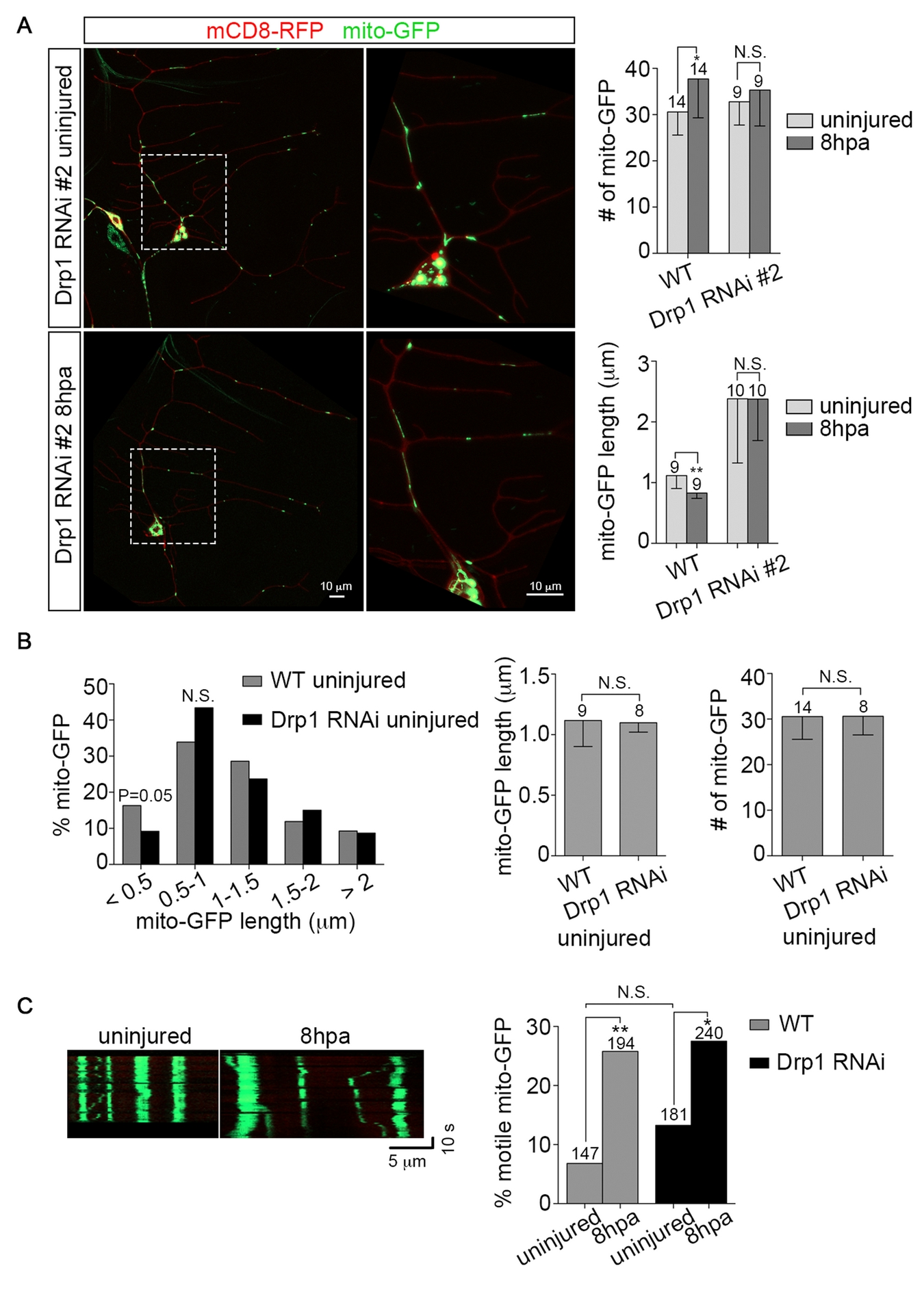

Supplement: S1 Fig — (A) Left, images of mitochondria in Drp1 RNAi #2-expressing neurons before and 8h post axon injury. mito-GFP and mCD8-RFP mark mitochondria and the cell membrane, respectively. Right, quantification of the average length and number of mitos in Drp1 RNAi #2 neurons is shown. The numbers of neurons analyzed are indicated above the bars. Statistical significance was determined with a t test. Error bars are SD. (B) The length distribution, average length and number of mitochondria are compared between WT and Drp1 (#1) RNAi neurons. Data of uninjured neurons from Fig 2B–2D’ were regraphed here to give a side-by-side comparison. (C) Left, kymographs of mito-GFP in the dendrites of wide-type neurons before and 8h post axon injury are shown. The X- and Y-axes represent distance and time, respectively. Right, quantification of mitochondrial motility is graphed. The numbers on the graph bars are the total numbers of mitochondria analyzed. Data were obtained from over six neurons for each genotype. Statistical significance was determined with a Fisher’s exact test. * p<0.05, ** p<0.01. (JPG) [file pgen.1006503.s001.jpg]

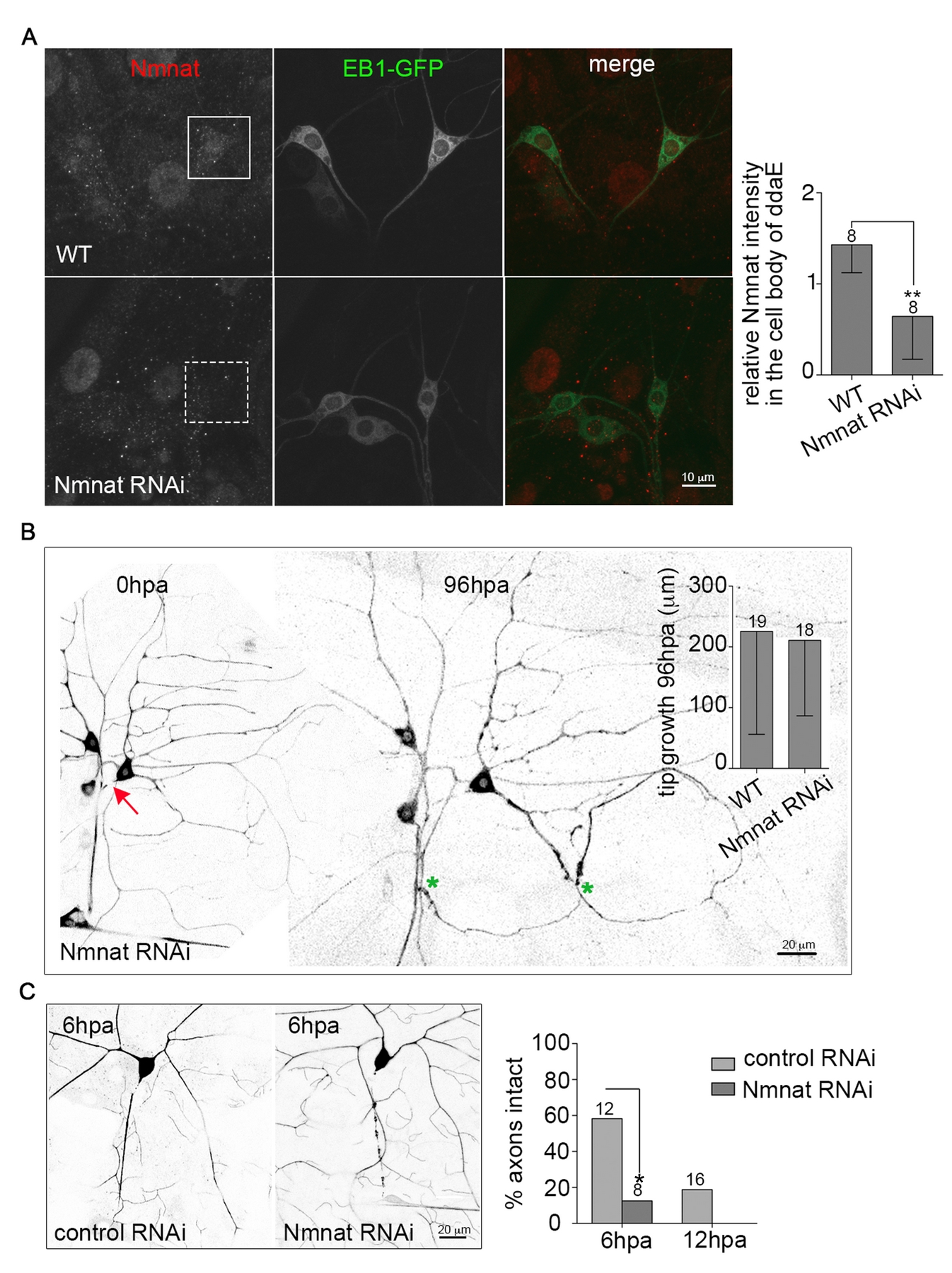

Supplement: S2 Fig — (A) Left, WT larvae and larvae in which Nmnat RNAi was expressed in several sensory neurons under control of 221-Gal4 were fileted, fixed and immunostained for endogenous Nmnat; for more information see S1 Methods. EB1-GFP was used as a cell marker. The squares outline ddaE cell bodies. Right, quantification of Nmnat intensity is shown. n = 8 neurons from 3 fillets. ** p<0.01, determined by an unpaired t test. Error bars are SD. (B) Axon regeneration was assayed in Nmnat RNAi neurons after proximal axotomy. Neurons were labeled with EB1-GFP driven by 221-Gal4. The red arrow shows the site where the axon was cut. The green stars indicate the tips of the dendrite that was converted into a regenerating axon after injury. The numbers of neurons analyzed are indicated above the bars. Statistical significance was determined by an unpaired t test. Error bars represent SD. (C) Axon degeneration was assayed in neurons expressing a control RNAi (Rtnl2) or Nmnat RNAi. Axons were severed at 0h, and then their integrity was assayed 6h and 12h later. Statistical significance was calculated with a Fisher’s exact test and * indicates p<0.05. (JPG) [file pgen.1006503.s002.jpg]

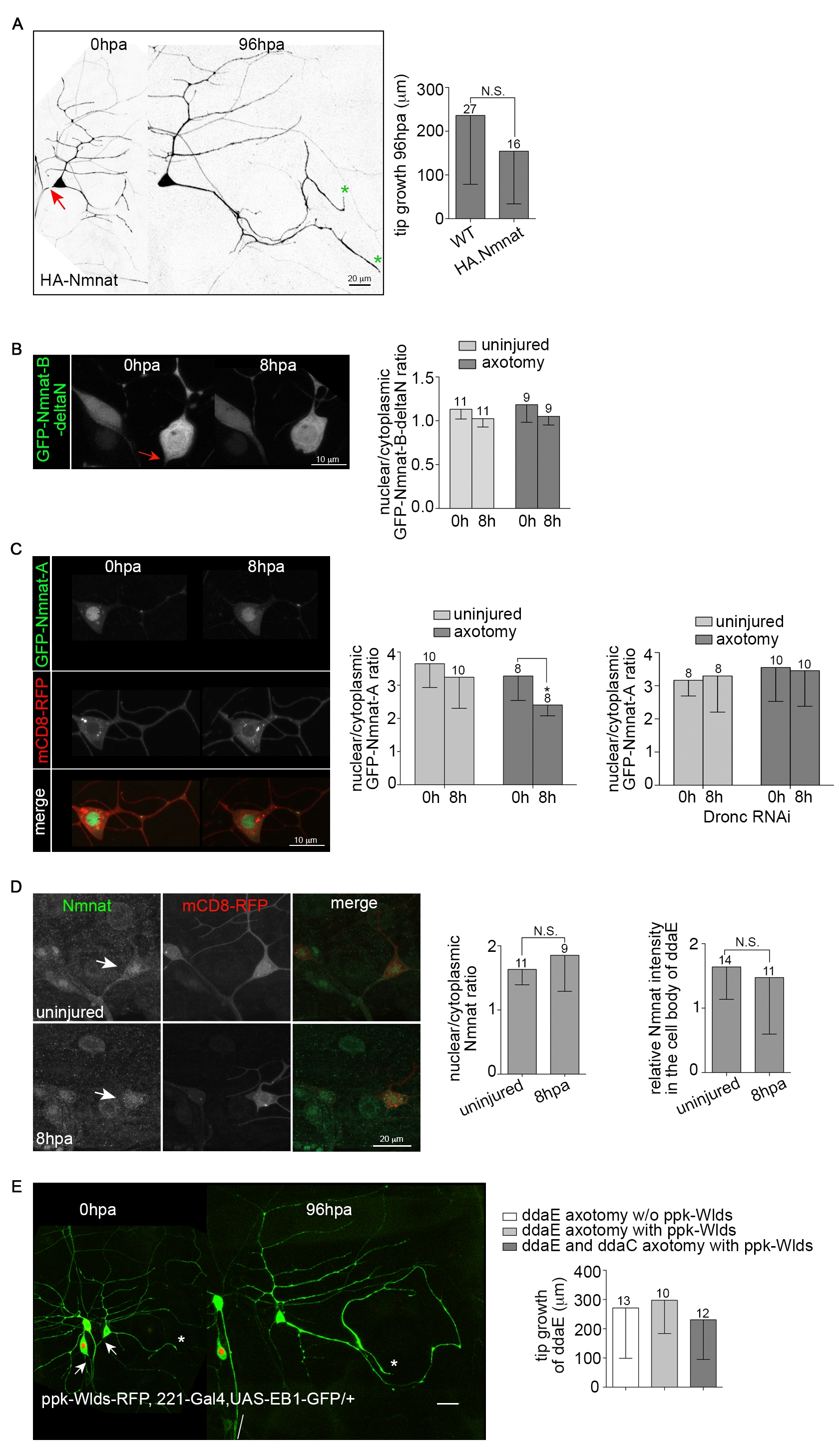

Supplement: S3 Fig — (A) Left, the axon regeneration assay was performed in UAS-HA-Nmnat and EB1-GFP-expressing neurons. The red arrow indicates the site of axon injury. The green stars indicate the tips of the dendrite that was converted to an axon at 96hpa. Right, quantification of the average length of regeneration is shown. The numbers of neurons analyzed are indicated above the bars. An unpaired t test was used to determine statistical significance. Error bars represent SD. (B) Left, GFP-Nmnat-B-deltaN was expressed in ddaE neurons under the control of 221-Gal4. The ratio of fluorescence intensity in the nucleus and cytoplasm was compared right after axotomy (0hpa) and 8hpa; see S1 Methods. The numbers of neurons analyzed are indicated on the bars. Statistical significance was determined by a paired t test. Error bars represent SD. (C) Left, GFP-Nmnat.A in ddaE neurons was imaged 0hpa and 8hpa. mCD8-RFP was co-expressed to mark cell membrane. Middle, quantification of the nuclear/cytoplasmic ratio of GFP intensity in WT neurons is shown. Right, quantification of the nuclear/cytoplasmic ratio of GFP intensity in Dronc RNAi neurons is shown. The numbers of neurons analyzed are indicated above the bars. Statistical significance was determined by a paired t test. Error bars represent SD. * p<0.05. (D) Left, endogenous Nmnat was stained in larval fillets from animals expressing mCD8-RFP in class I neurons; the ddaE neuron is indicated with arrows. Uninjured ddaE neurons or neurons 8h after axon severing are shown. Middle, quantification of the nuclear/cytoplasmic ratio of endogenous Nmnat is shown. Right, quantification of overall Nmnat intensity in the cell body of ddaE neurons is shown. The numbers of neurons analyzed are indicated on the bars. Data were obtained from 3 to 4 fillets. An unpaired t test was used to determine if any differences were significant. Error bars, SD. (E) Left, ddaC and ddaE neurons were labeled with EB1-GFP under the control of 221-Gal4. A persistent axon stu [file pgen.1006503.s003.jpg]
