# Supplementary material for: Mitochondria and Caspases Tune Nmnat-Mediated Stabilization to Promote Axon Regeneration
Source: PLoS Genet. 2016 Dec 6;12(12):e1006503. doi: 10.1371/journal.pgen.1006503 (PMC5173288; doi:10.1371/journal.pgen.1006503)
Supplement: S1 Methods — Methods including Nmnat immunofluorescence, analysis of mitochondrial motility, and generation of ppk-Wlds-td flies are described. (DOCX) [file pgen.1006503.s004.docx]

**S1 Methods**

Immunofluorescence of endogenous Nmnat

Third instar larvae expressing EB1-GFP or mCD8-RFP driven by 221-Gal4 were dissected in Schneider’s media and fixed in 4% paraformaldehyde (PFA) for 45 min, incubated in blocking solution (0.2% Triton X-100, 10 mM glycine, 1% BSA in PBS) for 10min. Filets were incubated with anti-Nmnat polycolonal antibody generated in guinea pig [35] diluted 1:1000 in blocking solution overnight, washed in blocking solution for 1h, and incubated with Rhodamine-conjugated goat anti-guinea pig antibody (1:50, Jackson ImmunoResearch Laboratories Inc.), or DyLight488-conjugated goat anti-guinea pig antibody (1:400, Jackson ImmunoResearch Laboratories Inc.) at room temperature for 1h. The average Nmnat intensity was measured in the cell body, nucleus and cytoplasm of ddaE neurons defined by EB1-GFP or mCD8-RFP fluorescence and was normalized to background.

Mitochondrial motility

Imaging was performed using a 63x objective and 2x zoom on a Zeiss LSM510 confocal microscope. Mitochondria in all dendrite branches in this field were quantified. Motility was calculated by counting the number of mitochondria that move over 1μm along dendrites in a one-minute imaging window. Kymographs were generated using the reslice tool in ImageJ with 1 pixel spacing.

Axon degeneration

mCD8-GFP, RNAi, and dicer2 transgenes were expressed in ddaC sensory neurons under the control of ppk-Gal4. Axons were axotomized closed to the cell body. The continuity of injured axons was determined 6 and 12h post injury. Axons that did not have morphological changes from uninjured ones were considered intact.

GFP-Nmnat levels

GFP-Nmnat-A and B-deltaN were expressed in ddaE neurons together with mCD8-RFP under the control of 221-Gal4. The average GFP intensity was measured in the cell body, nucleus and cytoplasm using Photoshop measurement tools, and was normalized to background. The normalized GFP intensity was used to calculate nuclear/cytoplasmic ratios.

Generation of ppk-Wlds-td flies

The coding sequence of Wlds was amplified from genomic DNA isolated from UAS-Wlds flies [37] with the forward primer 5’-ACTTGCAGATCTCAAAACATGGAGGAGCTGAGCGCTGAC-3’ containing a Bgl2 restriction site, and the reverse primer 5’-CATCGAACTAGTCAGAGTGGAATGGTTGTGCTT-3’ containing a Spe1 restriction site. The pCasper-ppk-EGFP plasmid was used as a PCR template for the ppk promotor with the forward primer 5’-ACATGCATGCAAGAGTTGGCAACAGGAG-3’ including an SphI restriction site, and the reverse primer 5’-CCGGAATTCGTACCCTAGAGGATCAGC-3’containing an EcoRI site. The PCR products of ppk promotor and Wlds were cloned into the vector pUAST::tdC. The final construct was injected into fly embryos to generate transgenic fly lines.
